# Experimental and Simulation Study on Residual Stress of Pure Copper Welded Joint by Laser Shock Peening

**DOI:** 10.3390/ma18174088

**Published:** 2025-09-01

**Authors:** Yandong Ma, Siwei Li, Yang Tang, Yongkang Zhang

**Affiliations:** 1 School of Electromechanical Engineering, Guangdong University of Technology, Guangzhou 510006, China; lswuot@163.com (S.L.); ty24012215@163.com (Y.T.); zykseu@163.com (Y.Z.); 2 Jiangsu Hengli Hydraulic Co., Ltd., Changzhou 213100, China; 3 Guangdong Provincial Key Laboratory of Advanced Manufacturing Technology of Marine Energy Equipment, Guangdong University of Technology, Guangzhou 510006, China

**Keywords:** laser shock peening, pure copper, TIG welding, butt-welded joint, residual stress

## Abstract

To accurately assess the residual stress distribution on the superficial layer of the weld for a pure copper butt-welded joint after laser shock peening (LSP), a coupled model was established by integrating experimental measurements with numerical simulations. This model simulates both the tungsten inert gas (TIG) welding process of pure copper and the subsequent LSP treatment applied to the weld. On this basis, the effects of the spot overlapping rate, number of impact layers, and pulse width on the weld residual stress profile were evaluated via multi-point LSP simulations. The findings imply that LSP converts the weld’s superficial residual stress from tensile to compressive, which verifies the accuracy of the simulations through the experimental data. Multi-point LSP numerical simulations demonstrate that elevating the spot overlapping rate and number of impact layers enhances the amplitude and affected depth of the surface compressive residual stress (CRS). A slight decrease in the CRS on the superficial layer of the weld was observed with an increase in pulse width. Compared with increasing the overlapping rate and pulse width, increasing the number of impact layers has a more significant strengthening effect. When the impact layer reached 3 times, the surface CRS reached −219.4 MPa, and the influence depth was 1.3 mm.

## 1. Introduction

In recent years, owing to the gradual saturation of wind power resources near the coast, the demand for offshore wind farms in deep sea areas has increased. High-voltage submarine cables are indispensable equipment, which are responsible for cross-sea power transmission. Limited by existing production technology, a single submarine cable is inadequate for ultra-long-distance power transmission. The flexible joint technology of submarine cables solves this technical problem through a segmented design and flexible connection [1]. Tungsten inert gas (TIG) welding is a very critical process in the production of submarine cable flexible joints. Before armoring, the copper conductors of the two submarine cables are connected through TIG welding to obtain better electrical conductivity. However, copper conductors may have defects during welding, including coarse grains, short fatigue life, and high-amplitude tensile residual stress (TRS) [2]. This is because pure copper has high thermal conductivity and a high linear expansion coefficient, resulting in high plastic deformation. Among these defects, residual stress is particularly important [3]. Different residual stress states may have different effects on the service life of components. TRS driven by fatigue load will increase the probability of crack nucleation and propagation near the weld, giving rise to accidental failure of the component. Compressive residual stress (CRS) may bring the opposite effect and prolong fatigue life [4,5,6]. Hence, improving the surface stress state after TIG welding of pure copper is of great significance for ensuring the safety of submarine cable transmission power.

Laser shock peening (LSP) is widely regarded as a promising method for strengthening weld surfaces. LPS employs a high-energy laser-induced shock wave to impart CRS on the metal surface, with depths reaching several millimeters [7]. Recent research has demonstrated that the CRS imparted by LSP is a critical factor governing the fatigue resistance of welded joints. Hatamleh [8] studied the effects of different LSP parameters on the crack propagation rate of friction stir welded (FSW) AA 2195 joints. The findings indicated that the fatigue crack propagation rate of the LSPed specimen was significantly reduced compared to that of the unpeened specimen, ascribed to the CRS introduced into the weld surface. Iordachescu et al. [9] reported the fatigue behavior of AA2024-T351 FSW joints after LSP. It was found that LSP inhibited pitting initiation and delayed stress corrosion cracking damage in welded joints by introducing beneficial CRS. Sadhu et al. [10] explored the influence of LSP on the surface residual stress and fatigue strength of FSW CuCrZr sheets. Their results demonstrated a notable gain in the penetration depth of CRS within the substrate, from 550 μm to 700 μm, as the number of LSP treatments rose from one to five. Despite the surface stress exhibiting no statistically significant variation, the fatigue durability of the FSW specimen after LSP could be extended by 70%. Wan et al. [11] found that LSP introduced a CRS of −460 MPa on the surface of a butt-welded joint of alloy 600 sheet after TIG welding, with the affected depth exceeding 1 mm and the average fatigue life increasing by 5.5 times. Tang et al. [2] explored the impact of double-sided simultaneous oblique LSP on the fatigue properties of pure copper welded joints under a single process parameter. They found that LSP formed a CRS of −155 MPa on the weld surface, accompanied by an increase in the fatigue limit from 83 MPa to 103 MPa. The above research confirms the feasibility of LSP to improve welded samples by introducing high-amplitude compression residual stress. Notably, achieving optimal residual stress distributions on weld surfaces remains experimentally difficult because of the inherent variability in LSP parameters. With the rapid development of computer technology, researchers have evaluated the influence of LSP parameters with the help of finite element simulation software [12,13,14]. Currently, numerical studies on LSP are predominantly focused on predicting the residual stress in aerospace substrate materials. However, a physical model of LSP on pure copper joints welded using TIG welding remains lacking due to the necessity of addressing the complexities of multi-physics coupling, which involves the thermo-elasto-plastic behavior from welding and the high-strain-rate dynamic plasticity induced by LSP.

In this work, the TIG welding process of pure copper is simulated using ABAQUS software, and the evolution of the temperature and residual stress are studied. According to the experimental data, the calculation results of heat and force using the welding model are verified. On this basis, explicit and implicit solution methods are used to establish the multi-point LSP finite element model of the weld, which is verified again. Subsequently, the residual stress profile of the weld surface under different LSP parameters is predicted. These findings provide a theoretical basis for using LSP to enhance the anti-fatigue manufacturing of copper conductor welds in submarine cable flexible joints.

## 2. Materials and Methods

### 2.1. Materials

A rolled rectangular plate of T2 pure copper with dimensions of 70 mm × 100 mm × 3 mm was employed as the welding material. It should be noted that the use of a rectangular plate instead of an applied cylindrical conductor was motivated by considerations of methodological feasibility. The performance of welded joints of high-voltage submarine cables mainly depends on the metallurgical response and microstructure evolution of materials under thermal-mechanical action. These basic physical processes are scale independent. The rectangular specimen provided a stable and repeatable surface for TIG welding and subsequent LSP due to its smooth and uniform geometric characteristics. It can not only significantly reduce the complexity of welding and LSP processing but also greatly facilitate the standardized implementation of residual stress field measurements, microstructure characterization, and mechanical property testing. Therefore, in the initial stage of the study, the intrinsic relationship between process parameters, microstructure, and properties was revealed with higher efficiency and controllability, which laid a foundation for understanding the LSP strengthening mechanism and optimizing process parameters.

### 2.2. Experimental Methods

**Figure 1:**
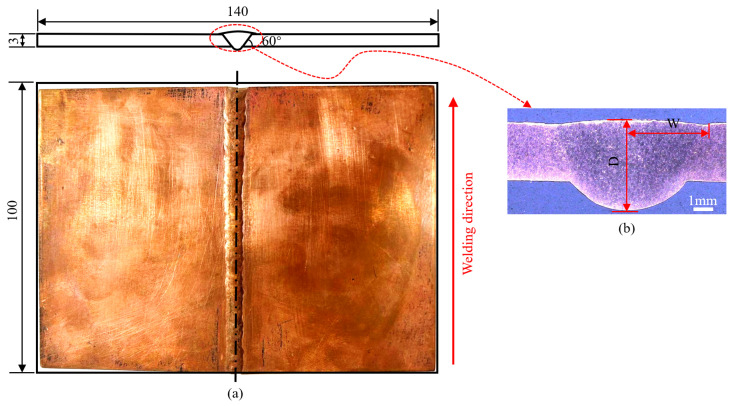
(**a**) Butt-welded joint specimens of pure copper (units in millimeters). (**b**) Macroscopic morphology of the weld.

The welding experiment was carried out according to the experimental scheme provided in Reference [2], and the welding parameters are presented in Table 1. Due to the high reflectivity of copper, the welding torch and the workpiece were approximately 80° during manual welding to avoid pure copper reflection. The pure copper sheet was tightly pressed onto a copper liner with good thermal conductivity. The physical object after welding is shown in Figure 1.

**Figure 2:**
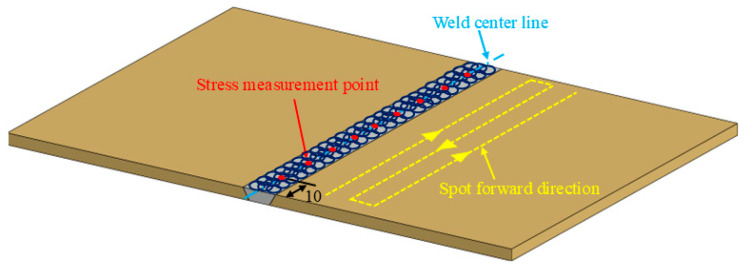
Schematic of the laser-forward direction and stress measurement of the weld.

Before LSP treatment, the weld reinforcement height was removed by mechanical processing. Then, the weld surface was polished with #800 and #1000 sandpapers, in turn. A layer of 0.1 mm black tape was pasted on the surface as the absorption layer. A confinement layer consisting of a ~2 mm film of deionized water was positioned over the absorption layer. The PROCUDO200 laser peening system (LSP Technologies, Inc., Dublin, OH, USA) was used to strengthen the weld, with its laser characteristics detailed in Table 2. The forward direction of the spot is depicted in Figure 2. The parameters employed in the LSP process are listed in Table 3. Residual stress measurements were performed along the weld centerline at 10 mm intervals using the sin^2^Ψ method with an XL-640 X-ray diffractometer. Surface stresses were characterized directly, while depth profiles were obtained through incremental material removal in 0.1 mm steps. All measurements were conducted in triplicate and averaged to ensure statistical reliability. The specific test parameters are detailed in Reference [2], and the corresponding measurement locations are illustrated in Figure 2.

### 2.3. Modeling Methods

**Figure 3:**
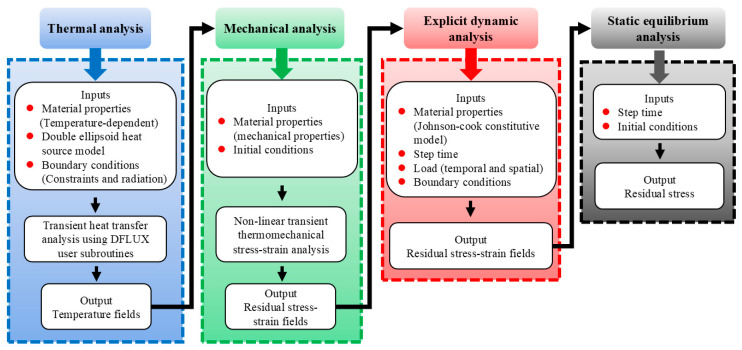
The flow chart of welding and LSP coupling numerical simulation.

The modeling idea of this work was divided into four stages, as outlined in Figure 3. Firstly, according to the experiment method of welding in Reference [2], the temperature field of the pure copper TIG welding process was modeled to compute the thermal history and distribution. Secondly, the temperature history was employed as a thermal load to analyze the welding stress field. Then, the stress field of the welding simulation served as the initial state of the LSP model, and the multi-spot dynamic solution was carried out. Finally, the dynamic analysis results were input into the ABAQUS 2022/Standard module as the initial conditions for springback analysis, and the final residual stress distribution was obtained.

The TIG welding process is a complex coupling field that integrates multiple factors, such as heat, force, deformation, and material phase transition. In this study, the following assumptions were made for the welding process to streamline the model and enhance computational efficiency:

- Suppose that the liquid metal of the molten pool is an incompressible Newtonian fluid, which is manifested as laminar flow.
- The molten pool exhibits a flat upper surface morphology.
- For the force driving the flow of liquid metal in the weld pool, only buoyancy, surface tension, and electromagnetic force are considered. The influence of positive pressure caused by arc pressure and surface curvature is ignored.
- The distribution of current density and heat flux of the welding arc is a double-ellipsoid heat source.
- The thermophysical properties of the target only change with temperature.

#### 2.3.1. Heat Conduction Analysis

The TIG welding process is a typical non-linear transient heat conduction process, and the governing partial differential equation for its temperature field is expressed as follows [15,16]:

(1) $$\rho C_{p}\frac{\partial T}{\partial t}=\frac{\partial }{\partial x}\left(\lambda_{x}\frac{\partial T}{\partial x}\right)+\frac{\partial }{\partial y}\left(\lambda_{y}\frac{\partial T}{\partial y}\right)+\frac{\partial }{\partial z}\left(\lambda_{z}\frac{\partial T}{\partial z}\right)+Q_{v}$$

where ρ denotes the material density, $C_{p}$ denotes the specific heat capacity, T denotes the temperature at the spatial coordinates $\left(x,y,z\right)$, and t denotes the time. $\lambda_{x}$, $\lambda_{y}$, and $\lambda_{z}$ are the thermal conductivities of the material along the x, y, and z directions, respectively. $\lambda =\lambda_{x}=\lambda_{y}=\lambda_{z}$ is taken without considering the anisotropy of the material. $Q_{v}$ is the internal heat source, including the latent heat change caused by the phase change of the material, as well as the impact of stress and deformation on the temperature field. It should be pointed out that the actual thermophysical parameters of materials, such as ρ, $C_{p}$, and λ, are all functions of T, which is also a critical factor in the calculation of welding temperature.

To carry out the finite element calculation, the governing partial differential equation of heat conduction needs to be discretized into a matrix form [17], as follows:

(2) $$[C_{e}]\left\{\dot{T}\right\}+\left[K_{e}\right]\left\{T\right\}=\left\{Q_{v}\right\}$$

where $\left[C_{e}\right]$ denotes the element heat capacitance matrix, and $\left[K_{e}\right]$ denotes the element thermal conduction matrix.

**Figure 4:**
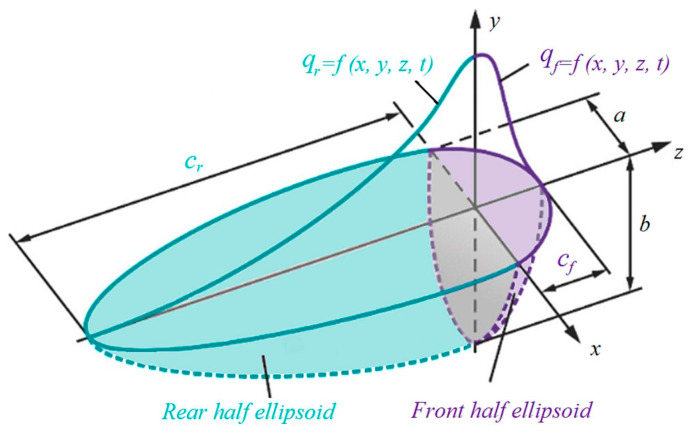
Double-ellipsoid heat source model [18].

In the actual welding process, due to the arc moving forward, the arc heat flux density is not symmetrical. The heating area in front of the arc is smaller than that behind the arc, and the energy density is significantly higher. Accordingly, the double-ellipsoid heat source model (see Figure 4 [18]) was implemented in this work to calculate the temperature field of pure copper TIG welding.

In the laboratory system of coordinates $\left(x,y,z\right)$, the profile of heat in the ellipse’s anterior half is expressed as follows [18]:

(3) $$q_{f}\left(x,y,z,t\right)=\frac{6\sqrt{3}f_{f}\eta UI}{abc_{f}\pi \sqrt{\pi }}\exp\left(-3\left(\frac{x^{2}}{a^{2}}+\frac{y^{2}}{b^{2}}+\frac{{\left(z-vt\right)}^{2}}{c_{f}^{2}}\right)\right),z>0$$

The thermal flux distribution across the elliptical posterior is given by [18]:

(4) $$q_{r}\left(x,y,z,t\right)=\frac{6\sqrt{3}f_{r}\eta UI}{abc_{r}\pi \sqrt{\pi }}\exp\left(-3\left(\frac{x^{2}}{a^{2}}+\frac{y^{2}}{b^{2}}+\frac{{\left(z-vt\right)}^{2}}{c_{r}^{2}}\right)\right),z<0$$

In Equations (3) and (4), U denotes the welding voltage (V), I denotes the welding current (A), η denotes the arc thermal efficiency, and a, b, $c_{r}$, and $c_{f}$ are the shape parameters of the double ellipsoid. a is the short semi-axis, b is the depth of the heat source, and $c_{f}$ and $c_{r}$ are the long semi-axes of the front and rear ellipsoids, respectively. $f_{f}$ and $f_{r}$ are the energy distribution coefficients of the anterior and posterior semi-ellipse, respectively. The two need to meet the following relationship:

(5) $$f_{r}+f_{f}=2$$

Since this study focused on the development and application of LSP process models, it lacked direct heat source calibration data. The following alternatives were adopted to ensure the effectiveness of the welding model as much as possible. Figure 1b shows the LT-3020 optical imaging system measuring the macro-section size of the weld pool. The weld possessed a width (W) of 4.1 mm and a depth (D) of 4.6 mm. According to the research results of Goldak [19], the heat source parameters governing the actual weld pool size are as follows: $c_{f}$ = a = 0.9 W; $c_{r}$ = 2 $c_{f}$; $f_{r}$ = 2 $f_{f}$; and b = 0.9 D. To minimize discrepancies between the simulated and measured weld pool geometry, as well as measurement error, the parameters of the heat source model were calibrated using an iterative trial-and-error process. Table 4 presents the finalized welding heat source parameters. The residual stress simulation results obtained by the heat source model parameters were compared with the experimental measurement results to verify the effectiveness of the welding model.

Furthermore, the welding process involves the workpiece melting and its subsequent solidification upon cooling. The energy changes during the solid–liquid phase transition have significant implications for the computation of the thermal and stress profiles in welding. The latent heat of phase change is therefore incorporated via the sensible heat capacity method, mathematically expressed as follows:

(6) $$\Delta H=\left(c^{*}-c\right)\left(T_{L}-T_{s}\right)$$

where ΔH is the phase change heat, $c^{*}$ denotes the equivalent hot melt, c denotes the average value of the specific heat capacity of the solid and liquid phases of the material, and $T_{L}$ and $T_{s}$ are the liquid and solid phase temperatures, respectively.

In the process of finite element calculations, except for the control equation, some boundary conditions describing the model’s initial condition are also needed. Initial conditions in the thermal analysis encompass two key parameters: the initial temperature of the specimen and the ambient temperature before welding begins. In order to facilitate the calculation, the initial temperature is usually regarded as the same as the ambient temperature when the influence of welding preheating on the temperature is not considered:

(7) $$T\left(x,y,z,t=0\right)=T_{\infty }$$

where $T_{\infty }$ denotes the ambient temperature.

The boundary conditions in the thermal model account for the heat transfer between the workpiece and the surrounding environment. Three different boundary conditions are usually considered [20].

A prescribed temperature is applied to the $\Gamma_{1}$ boundary, constituting the first type of temperature boundary condition:

(8) $$T=T\left(\Gamma_{1},t\right)$$

The second boundary condition is used to describe the given heat flux density (W·m^−2^) on the boundary:

(9) $$\lambda \frac{\partial T}{\partial n}=T\left(\Gamma_{2},t\right)$$

The third type of boundary condition is used to characterize convective heat transfer at the interface:

(10) $$\lambda \frac{\partial T}{\partial n}=h\left(T_{0}-T\right)$$

In Equations (8)–(10), h denotes the convective heat transfer coefficient (W·m^−2^·K^−1^). Under natural convection conditions, $T_{0}$ is the ambient temperature. Under forced convection, $T_{0}$ denotes the adiabatic wall temperature of the boundary layer, and $\Gamma_{1}$ and $\Gamma_{2}$ indicate that the distribution of temperature T on the boundary may change with position. In this work, only the second and third boundary conditions were considered in the simulation of the welding temperature field.

#### 2.3.2. Mechanical Analysis

The temperature history served as the input for the subsequent mechanical analysis [21]. The elements in the model structure satisfied the following equilibrium equation [20]:

(11) $${\left\{dF\right\}}^{e}+{\left\{dR\right\}}^{e}={\left[K\right]}^{e}{\left\{d\delta \right\}}^{e}$$

where ${\left\{dF\right\}}^{e}$ denotes the stress increment on a certain node, ${\left\{dR\right\}}^{e}$ denotes the stress increment generated by the initial strain equivalent node affected by temperature, ${\left[K\right]}^{e}$ is the stiffness matrix, and ${\left\{d\delta \right\}}^{e}$ denotes the displacement increment of a certain node. ${\left[K\right]}^{e}$ and ${\left\{dR\right\}}^{e}$ can be calculated as follows:

(12) $${\left[K\right]}^{e}={\int \left[B\right]}^{T}\left[D\right]\left[B\right]dV$$

(13) $${\left\{dR\right\}}^{e}={\int \left[B\right]}^{T}\left\{C\right\}dTdV$$

where $\left[B\right]$ denotes the correlation matrix of the strain vector and the node displacement vector of the selected element, $\left[D\right]$ is the elastic matrix, and $\left\{C\right\}$ denotes the temperature-related vector.

When the material is in the elastic stage, $\left[D\right]={\left[D\right]}_{e}$ and $\left\{C\right\}={\left\{C\right\}}_{e}$, and in the yield state, $\left[D\right]={\left[D\right]}_{ep}$ and $\left\{C\right\}={\left\{C\right\}}_{ep}$.

By using ${\left[D\right]}_{e}$ or ${\left[D\right]}_{ep}$ and ${\left\{C\right\}}_{e}$ or ${\left\{C\right\}}_{ep}$ to replace $\left[D\right]$ and $\left\{C\right\}$ in Equations (12) and (13), respectively, the element stiffness matrix and equivalent node load can be obtained. Combined with the total stiffness $\left[K\right]$ and the total load vector $\left\{dF\right\}$, the equilibrium equations of the whole model structure can be obtained, as follows:

(14) $$\left[K\right]\left\{d\delta \right\}=\left\{dF\right\}$$

(15) $$\left[K\right]={\sum \left[K\right]}^{e}$$

(16) $$\left\{dF\right\}=\sum \left({\left\{dF\right\}}^{e}+{\left\{dR\right\}}^{e}\right)$$

The simulation of welding stress and strain usually excludes the influence of external construction forces. The force in each element is a self-balanced system, that is, it is in a balanced state. At this time, $\sum \left({\left\{dF\right\}}^{e}\right)=0$ is substituted into Equation (16), and $\left\{dF\right\}={\sum \left\{dR\right\}}^{e}$ can be obtained.

#### 2.3.3. Establishment of the Model

**Figure 5:**
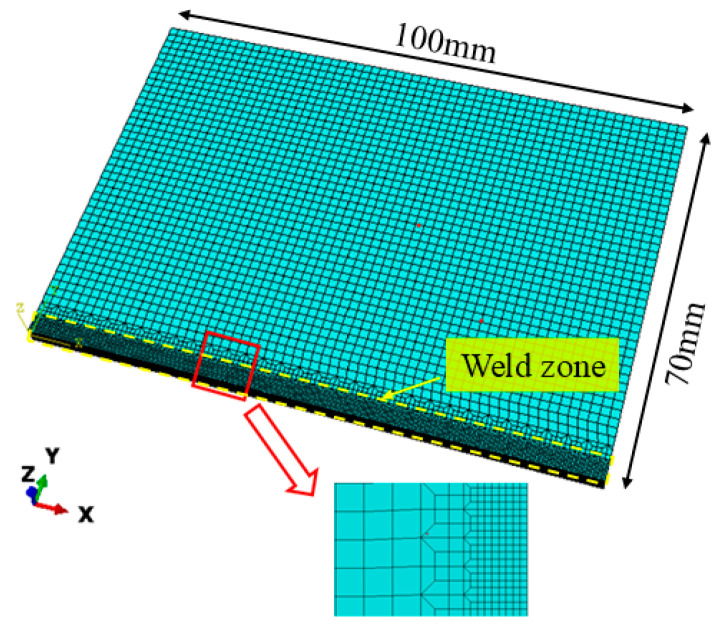
Mesh model of pure copper butt weld.

Since the butt-welded joint specimen was approximately symmetrical along the weld center, a half model was established to reduce the computational expense, as illustrated in Figure 5. The model was divided into non-uniform grids. A fine mesh of 0.4 mm × 0.4 mm × 0.2 mm was applied in the weld zone to capture more accurate temperature and stress gradients. A non-uniform mesh was applied to areas away from the weld, following a linear grading rule, with the coarsest grid dimensions set to 1.5 mm × 1.5 mm × 1 mm. Therefore, the computation model had a total of 29,044 elements, and the element type of the grid was 8-node linear heat conduction element (DC3D8).

**Figure 6:**
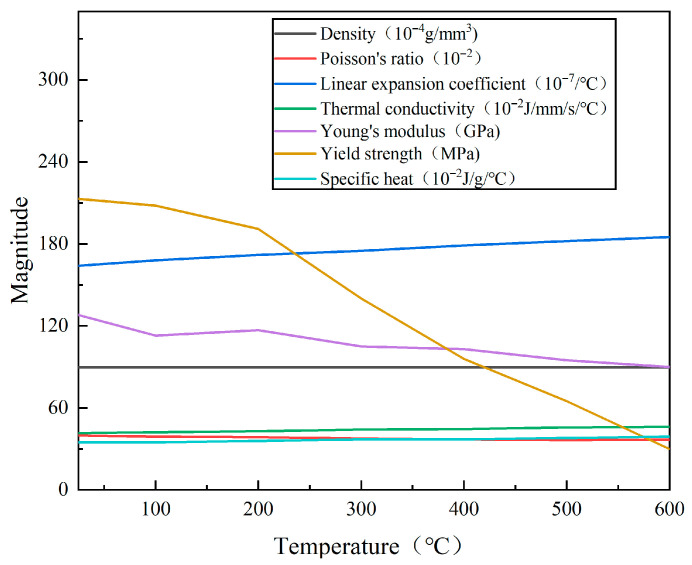
Thermal physical parameters of T2 pure copper [22].

Heat source movement along the weld was obtained using the Dflux subroutine compiled in Fortran language. The element birth and death technique was implemented via a Python (version 3.9.12) program to calculate the weld activation process. The whole weld was divided into 50 analysis steps, and 5 layers of each step were activated. The time of each step was set to 1 s, the total welding time was 50 s, and the welding speed was 2 mm/s. In the temperature field calculation model, the initial condition was defined as a uniform temperature of 25 °C. The thermal boundary conditions were set as follows: a convective heat transfer coefficient of 25 W/(m^2^·K), a radiation coefficient of 0.7, a Stefan–Boltzmann constant of 5.67 × 10^−8^ W/(m^2^·°C), and an absolute zero of −273.15 °C. In accordance with the welding experiments, a simplified clamping constraint was adopted, that is, the two ends of the model were completely constrained, and the side of the weld was symmetrically constrained. The thermophysical properties of pure copper at different temperatures are shown in Figure 6 [22].

## 3. Results and Discussion

### 3.1. Thermal Analysis

**Figure 7:**
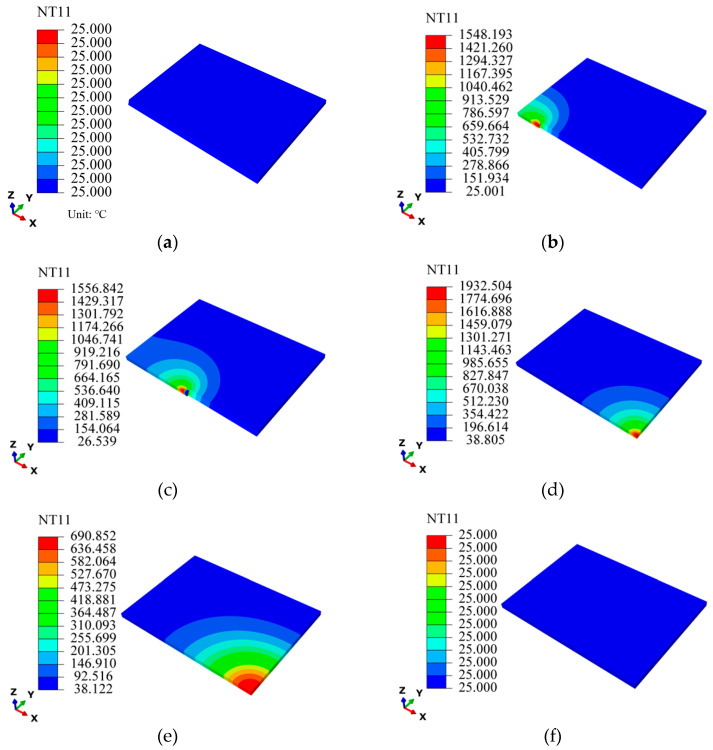
Temperature distribution changes of welded specimen with time: (**a**) t = 0 s; (**b**) t = 10 s; (**c**) t = 25 s; (**d**) t = 50 s; (**e**) t = 94 s; (**f**) t = 487 s.

A cloud diagram of the computed temperature field is shown in Figure 7. The temperature fields at 0 s, 10 s, 25 s, 50 s, 94 s, and 487 s are given, respectively, to analyze the variation in the temperature field during TIG welding of pure copper. This figure depicts the evolution of the welding thermal field ahead of and behind the advancing heat source, which exhibits an elliptical isothermal profile with a pronounced thermal gradient from its core to the periphery. The overall temperature field exhibits symmetry about the weld centerline, which is consistent with the thermal distribution law of the double-ellipsoid model. When t = 0 s, as illustrated in Figure 7a, the whole model temperature is room temperature. In Figure 7b, the temperature at the beginning position of the weld at t = 10 s increases rapidly, and the temperature at the center of the weld is up to 1548 °C, which exceeds the melting point of T2 pure copper. The molten pool penetrates the entire weld, indicating that the weld is fully welded. As Figure 7c indicates, the maximum temperature of the weld center reaches 1557 °C at t = 25 s. At the end of welding, the peak temperature at the weld center is 1933 °C, as shown in Figure 7d. This suggests that the temperature of the entire weld gradually increases over time, with a more significant rise occurring during the later stage of the welding process. This is because in the initial stage of welding, most of the heat generated by the arc is used to preheat the entire welded specimen. Moreover, the temperature at the weld center is relatively low at the initial stage owing to the high thermal conductivity of pure copper. In the later stage of welding (t = 94 s), the heat accumulation effect occurs due to the continuous absorption of heat by the welded specimen, so the temperature of the weld center increases significantly, as shown in Figure 7e. At this time, the peak temperature of the weld drops to 691 °C. When cooled to 487 s, the temperature of the entire welded specimen is reduced to room temperature, as shown in Figure 7f.

### 3.2. Stress Field Analysis

The directional heat input along the weld seam results in significant longitudinal thermal expansion and contraction, ultimately giving rise to high TRS upon cooling. These stresses intensify stress concentration within the weld area, resulting in crack formation and growth along the weld direction [23]. Thus, the longitudinal residual stress is used as a key indicator to evaluate the welding quality and structural reliability of the weldment.

**Figure 8:**
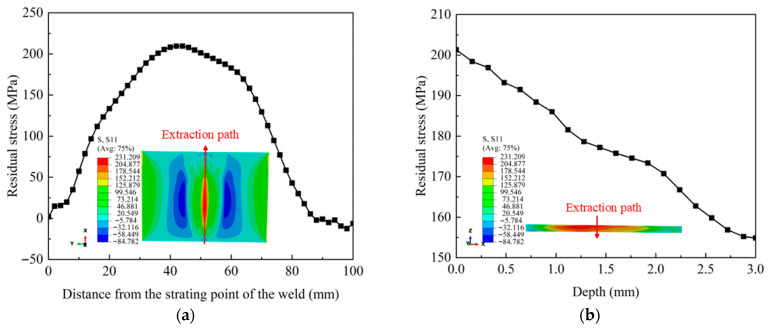
Stress cloud diagram of pure copper after welding: (**a**) surface; (**b**) depth.

Figure 8 exhibits the stress cloud diagram and distribution curve of the weld surface. In this figure, the CRS appears in the area far from the weld and is symmetrically distributed about the weld. The reason is that the weld and its surrounding zone undergo thermal expansion during the welding process, but the ends of the welded specimen are restrained longitudinally. To achieve stress equilibrium, the CRS is generated in the area distal to the weld. As observed in Figure 8a, the maximum TRS is at about 40~50 mm from the starting point of the weld, with a value of 231.2 MPa. The residual stress value at both ends of the weld is 0. A TRS field exists throughout the weld thickness, where the stress value gradually decreases as the depth increases (see Figure 8b).

### 3.3. Verification of Simulation Results for Welding

**Figure 9:**
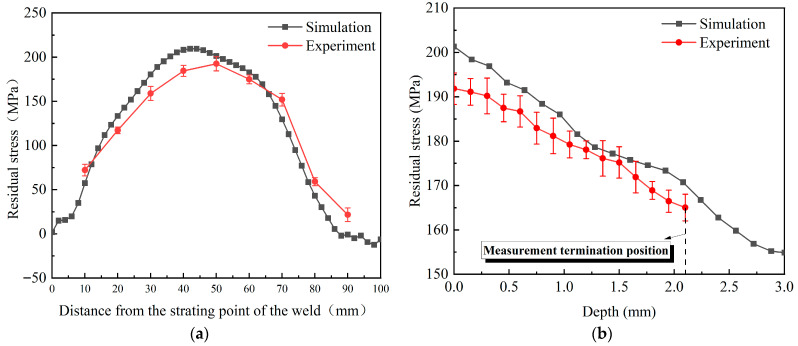
Comparison of experimental and numerical results of welding residual stress: (**a**) surface; (**b**) depth.

Figure 9 compares the computed and measured stress results at the weld centerline. As Figure 9a indicates, the measured residual stress profile on the weld surface corresponds closely to the simulated value. The peak values of the computed and measured TRS reach 209.5 MPa and 192.5 MPa, respectively, showing an error of 8.8% between the two values. However, the maximum position of the TRS between the two is different. The simulated value occurs at 40 mm~45 mm from the starting point of the weld, while the measured value is at 45 mm~50 mm. In Figure 9b, the TRS reaches its maximum at the weld surface and progressively decreases with depth, while the experimental and numerical profiles show converging distribution patterns in subsurface regions. In summary, it can be considered that the established numerical simulation model of pure copper TIG welding is effective. The discrepancy between simulated and experimental outcomes arises from multiple contributing factors, including constraint conditions, mesh size, material thermophysical properties, and the radiative heat transfer coefficient.

### 3.4. Verification of Simulation Results for LSP

After welding, the element type of the model was modified to C3D8R to avoid the mesh distortion caused by the high strain rate of LSP. The multi-point LSP numerical model for the post-welding condition was established by introducing the welding stress through a predefined field as the initial stress condition. The peak pressure of the shock wave within a single circular spot was calculated according to the physical model proposed by Fabbro [24]:

(17) $$P_{\max}(\mathrm{GPa})=0.01\sqrt{\frac{\alpha }{2\alpha +3}}\sqrt{I_{0}(\mathrm{GW}/cm^{2})}\sqrt{Z(g\cdot cm^{-2}\cdot s^{-1})}$$

(18) $$\frac{2}{Z}=\frac{1}{Z_{t\arg et}}+\frac{1}{Z_{water}}$$

(19) $$I_{0}=\frac{E}{S\tau }$$

where α is the fraction of internal energy converted into heat energy (α = 0.1 [25]), $I_{0}$ denotes the laser power density, Z denotes the equivalent acoustic impedance, and $Z_{t\arg et}$ = 1.46 × 10^6^ g/(cm^2^·s) [25] and $Z_{water}$ = 1.14 × 10^6^ g/(cm^2^·s) [25] are the acoustic impedance of the target material and the water confinement layer, respectively. E, S, and τ are the laser pulse energy, spot area, and pulse width, respectively.

It is generally accepted that the actual action time of LSP-induced pulse pressure may be two to three times longer than that of the laser pulse [26]. Hence, the total pressure duration of a single LSP process was set to 60 ns, and the history of pulse pressure with time was simplified to a triangular ramp in this simulation study.

Considering that the energy output of the laser beam employed in this work follows a flat-top spatial distribution, the pressure distribution in the LSP area can be expressed as follows:

(20) $$P\left(x,y,t\right)=P\left(t\right)\exp\left[-2{\left(\frac{\sqrt{x^{2}+y^{2}}}{R}\right)}^{10}\right]$$

where $\left(x,y\right)$ and R are the loading position and radius of a single spot LSP process, respectively. P(t) is the function of shock wave pressure with respect to time t. The pressure pulse and spot scanning sequences were performed in ABAQUS 2022/Explicit using a VDLOAD subroutine. More details of the LSP modeling work can be found in Reference [27]. The Johnson–Cook (J–C) constitutive model and essential mechanical properties of pure copper are listed in Table 5 [25].

**Figure 10:**
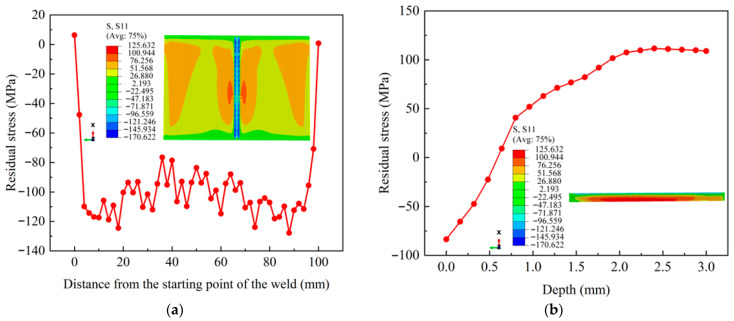
The stress simulation results of the weld after LSP with 5.5 J laser energy: (**a**) surface; (**b**) depth.

Figure 10 depicts the stress distribution across surface and subsurface regions of the weld processed by multi-point LSP. The laser peening parameters employed in the simulation were maintained with those in the experiments. The stress contour plot in Figure 10a demonstrates a complete shift from tensile to compressive stress states. Fluctuations in CRS are observed along the weld surface, ranging from −127.8 MPa to −76.5 MPa. After LSP, a CRS-affected layer of about 0.58 mm is formed on the weld surface, showing monotonically decaying stress values with increasing depth, as shown in Figure 10b.

**Figure 11:**
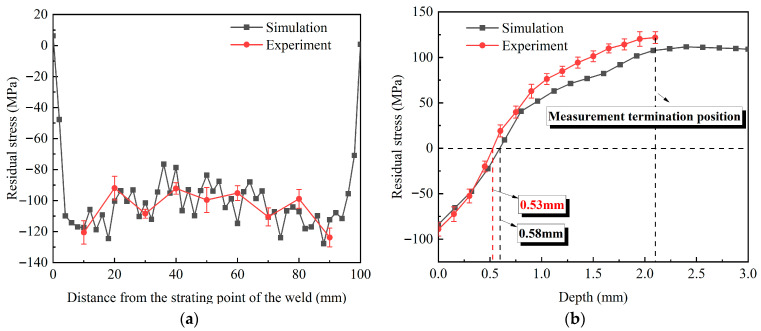
Comparison between simulated and experimentally measured residual stresses along the weld centerline after LSP: (**a**) surface; (**b**) depth.

Figure 11 compares the computed and experimental stresses of the weld after LSP. A strong correlation is observed between the simulated and measured stresses, both at the surface and through the depth. A maximum surface CRS of −121.6 MPa is recorded, which differs by 5% from the simulated results. The affected layer of CRS is 0.53 mm, and the error is 9.4%. The evaluation results confirm the validity of the model.

### 3.5. Influence of LSP Parameters on the Distribution of Residual Stress in the Weld

#### 3.5.1. Overlapping Rate

**Figure 12:**
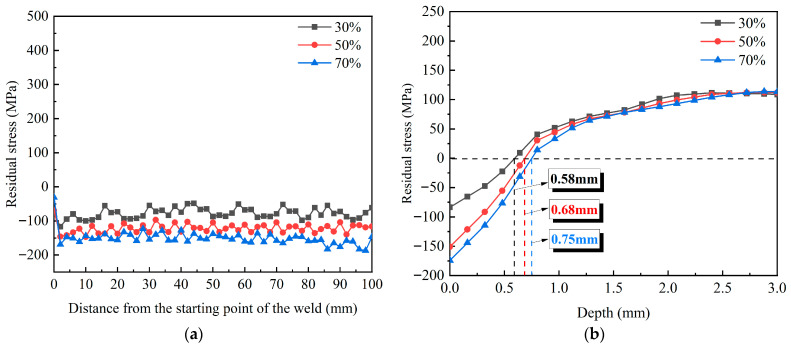
Residual stress distribution under different overlap rates: (**a**) surface; (**b**) depth.

In the case of other laser process parameters remaining unchanged, the LSP numerical simulation of the weld was carried out using overlapping rates of 30%, 50%, and 70%, respectively. Figure 12 depicts the resulting residual stress distributions on the weld surface and along its depth. Figure 12a exhibits an increasing trend in the CRS on the weld surface as the overlapping rate is increased. At 30%, 50%, and 70% overlapping rates, the maximum CRS values are −127.8 MPa, −159.7 MPa, and −188.2 MPa, respectively. The figure also indicates that, with a decrease in the overlapping region, the compressive stress value of the surface fluctuates obviously. This is because when the circular spot irradiates the target surface, rarefaction waves generated at its periphery converge toward the center. This convergence induces reverse plastic deformation at the focal point, which consequently diminishes the residual stress [25]. Furthermore, the energy density in the overlapping area of the spots exceeds that of the non-overlapping area, so the CRS distribution is not uniform.

The in-depth residual stress of the weld caused by different overlapping rates is presented in Figure 12b. A similar pattern is observed in all three cases, with a gradual decay in CRS as the depth increases. This results from the attenuation of energy as the laser-induced shock wave propagates through the material. At overlapping rates of 30%, 50%, and 70%, the affected depths of the CRS are 0.58 mm, 0.68 mm, and 0.75 mm, respectively, indicating a positive correlation between the affected depth and the overlapping rate.

Hence, the increase in the non-overlapping area is the reason for the uneven distribution of surface CRS and the decrease in its affected depth. Since the dynamic yield strength of pure copper at high strain rates is significantly higher than its static value [28], the non-overlapping region cannot overcome this strength and produce a sufficiently deep plastic deformation layer. Therefore, the CRS layer is shallow. At the same time, a deep enough plastic deformation layer is formed in the overlapping region with multiple impacts. There is a huge difference in the degree of plastic deformation between the region and the non-overlapping region, which leads to uneven distribution of surface residual stress.

#### 3.5.2. Number of Impact Layers

**Figure 13:**
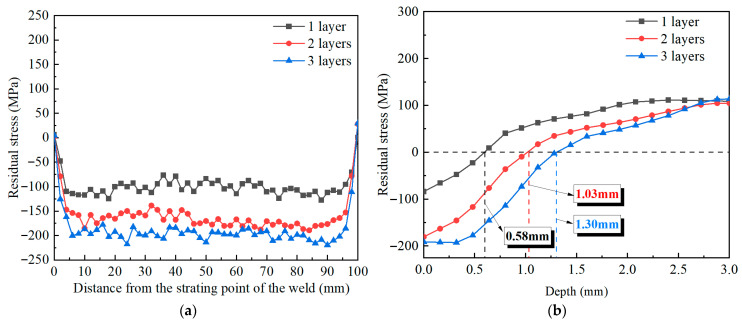
A comparison of residual stress for different numbers of impact layers: (**a**) surface; (**b**) depth.

Figure 13 compares the surface and in-depth residual stresses in the weld under different numbers of impact layers, with other parameters consistent with the experiments. In Figure 13a, the maximum CRS on the weld surface is −127.8 MPa after one impact. With the number of impact layers rising to 2 and 3, the maximum compressive stress values increase to −189.5 MPa and −219.4 MPa, respectively. The growth rates are 48.3% and 15.8%, respectively. Results demonstrate that the CRS of the weld surface rises progressively with a rising number of impact layers. When the number of impact layers is increased to 3, the rise in the maximum CRS is less pronounced compared to 2 layers, indicating a trend toward saturation in the surface residual stress. The affected depths corresponding to 1, 2, and 3 layers are 0.60 mm, 1.03 mm, and 1.30 mm, respectively, as shown in Figure 13b. After one impact, the laser-induced shock wave pressure exceeds the dynamic yield strength of the target, and the energy of the shock wave is rapidly absorbed by the surface of the target and produces elastic-plastic deformation. After two impacts, the superposition of the shock wave causes the accumulation of elastic-plastic deformation on the surface of the target, resulting in deeper plastic deformation. After three impacts, the plastic deformation in this region gradually saturates, and the target’s ability to absorb shock wave energy is gradually weakened. Owing to this mechanism, the in-depth residual stress exhibits a progressively saturation trend with more impact times.

#### 3.5.3. Pulse Width

**Figure 14:**
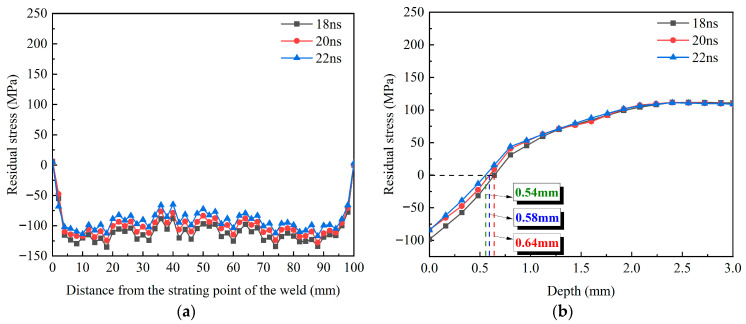
Residual stress distribution at varying pulse widths: (**a**) surface; (**b**) depth.

Figure 14 illustrates the surface and in-depth residual stresses of the weld under three different pulse widths. As depicted in Figure 14a,b, the CRS on the weld surface and its affected depth exhibit a slight decrease with increasing pulse width. At pulse widths of 18, 20, and 22 ns, the respective stress values are −135.3, −127.8, and −117 MPa, with corresponding affected depths of 0.64, 0.58, and 0.54 mm. Although a longer pulse width extends the shock wave duration, the concomitant decrease in laser power density results in a reduction of both the surface CRS and affected depth. In summary, compared with the lap rate and impact times, changing the pulse width has little effect on the strengthening effect of pure copper welds.

## 4. Conclusions

In this study, a coupled model for TIG welding of pure copper and subsequent LSP treatment of the weld was established. This model effectively addresses the limitations of conventional approaches in simultaneously accounting for the interaction between the initial welding-induced stress field and LSP-induced shock wave effects. The model was validated against experimental data. On this basis, the surface and in-depth residual stresses of the weld under different overlapping rates, impact times, and pulse widths were obtained. The study’s main outcomes are as follows:

- A coupled numerical model integrating the thermo-elasto-plastic behavior of pure copper TIG welding with the high-strain-rate dynamic plasticity of LSP is established. The error between the predicted residual stress and the experimental results is less than 9.4%.
- The TIG weld of pure copper was characterized by a high TRS, with a peak value of 231.2 MPa located along the weld centerline. Following multi-point LSP treatment at 5.5 J, a CRS field was achieved on the weld surface. An increase in the overlapping rate results in a corresponding rise in both the surface CRS on the weld and the affected depth.
- Whereas increasing the overlapping rate offers modest gains, increasing the number of impact layers markedly improves weld strengthening, yielding a CRS of −219.4 MPa and an affected depth of 1.3 mm after three impacts.
- Both the surface CRS and its affected depth exhibit a decreasing trend with increasing pulse width.

## Figures and Tables

**Table 1:** TIG welding parameters of pure copper [2].

Category	Parameter
Plate thickness (mm)	3
Voltage (V)	23
Current (A)	130
Speed (mm/s)	2
Welding wire diameter (mm)	2
Argon flow rate (L/min)	10

**Table 2:** The corresponding laser characteristics of the PROCUDO200 laser shot peening system.

Laser Characteristics	Category/Parameter
Laser type	Diode pumped pulsed YLF laser
Laser wavelength (nm)	1053
Repetition rate (Hz)	1~20
Maximal pulse energy (J)	10
Laser pulse duration (ns)	18~22

**Table 3:** LSP process parameters for the weld of pure copper.

Laser Pulse Energy (J)	Pulse Width (ns)	Spot Diameter (mm)	Overlapping Rate (%)	Layers of Impact	Incidence Angle (°)
5.5	20	2.5	30	1	90

**Table 4:** Related parameters of the welding heat source.

Parameter	U (V)	I (A)	η	*a* (mm)	*b* (mm)	$c_{f}$ (mm)	$c_{r}$ (mm)
Value	23	130	0.8	2.80	4.60	2.80	5.60

**Table 5:** Mechanical properties and J–C constitutive model of pure copper [25].

Density ρ (Kg/m^3^)	Elastic Modulus E/GPa	$\mathrm{Air}\;\mathrm{Temperature}\;T_{r}$/°C	$\mathrm{Melting}\;\mathrm{Point}\;T_{m}$/°C	Static Shear Strength A/MPa	Strain Hardening Modulus B/MPa	Strain Hardening Exponent n	Strain rate Sensitivity Coefficient C
8969	110	25	1083	50	312.4	0.3572	0.0438

## Data Availability

The original contributions presented in this study have been included in the article. Further inquiries can be directed to the corresponding author.

## Abbreviations

The following abbreviations are used in this manuscript:

TIG	Tungsten inert gas
LSP	Laser shock peening
